# Pilot study: molecular risk factors for diagnosing sporadic Parkinson's disease based on gene expression in blood in MPTP-induced rhesus monkeys

**DOI:** 10.18632/oncotarget.22348

**Published:** 2017-11-10

**Authors:** Liangqin Shi, Chao Huang, Qihui Luo, Yu Xia, Heng Liu, Like Li, Wentao Liu, Wenjing Ma, Jing Fang, Li Tang, Wen Zeng, Zhengli Chen

**Affiliations:** ^1^ Laboratory of Animal Disease Model, College of Veterinary Medicine, Sichuan Agricultural University, Chengdu, Sichuan 611130, China; ^2^ Key Laboratory of Animal Disease and Human Health of Sichuan Province, College of Veterinary Medicine, Sichuan Agricultural University, Ya'an, Sichuan 625014, China; ^3^ Sichuan Primed Biological Technology Co., Ltd, National Experimental Macaque Reproduce Laboratory, Ya'an, Sichuan 625014, China

**Keywords:** Parkinson’s disease, risk factor, monkey, blood

## Abstract

Clinical diagnosis of Parkinson's disease (PD) is characterized by the classical features of tremor, bradykinesia and rigidity, which are present only when more than 70%-80% degeneration of dopaminergic (DA) neurons in the substantia nigra. The lack of means for early diagnosis of PD has elicited interest in searching for its risk factors, which, by now, are almost obtained at a single time point in PD process, and little developing risk factors, obtained from completely normal situation to the onset or even advanced stage of PD in individual person which could monitor the progress of PD, are present. Here we have detected some potential factors in the blood of MPTP induced PD monkeys along with the progress of the disease. All the PD monkeys showed mild PD symptoms since the 9^th^ week and gradually reached a classic and stable parkinsonism stage at the 18^th^ week. Our results have found that the expression of Parkin, USP30, MUL1, PINK1, and LRRK2 significantly increased at 1^st^, 3^th^, 3^th^, 5^th^, and 8^th^ week respectively and remained high till the end; The expression of UCHL1 and TRIM24 significantly increased at the 1^st^ and 18^th^ week, respectively, then gradually decreased and significantly lower than normal value; DJ-1 showed significantly decreased since the 12^th^ week, while SNCA showed no significantly changed excepted at the 5^th^ week. And, the terminal results of whole blood were highly consistent with those of in SN. These results support that these genes change may as biomarkers to monitor the progress of PD, and may facilitate the development of biomarkers for early diagnosis.

## INTRODUCTION

As a progressive neurodegenerative disease with a prevalence of 1-2% in the population aged above 65 years [1–3], Parkinson's disease (PD) is characterized by the main features of tremor, bradykinesia and rigidity, which are present only when more than 70%-80% degeneration of dopaminergic (DA) neurons in the substantia nigra (SN) [4–6]. The profiles of gene expression, such as SNCA, Parkin, PINK1, LRRK2, UCHL1, TRIM24, MUL1, USP30, and DJ-1, have already been shown to be altered in the substantia nigra of PD patients, and may act as potential risk factors in PD diagnosis [2, 7–11]. Healy *et al* found that six mutations of LRRK2 were proven pathogenic to PD and the frequency of the common LRRK2 Gly2019 Ser mutation was 1% of sporadic PD patients and 4% of hereditary PD patients [2]. Maraganore's research found that ubiquitin carboxy-terminal hydrolase L1 (UCHL1) was a susceptibility gene for PD and a potential target for disease-modifying therapies [12]. However, these genes expression profiles could not be analyzed in brain during life and were mainly obtained in PD subjects accompanied with classic motor symptoms [13]. Similarly, clinic diagnosis is hard to made before these signs and symptoms appear. Therefore, a wide agreement is that preclinical risk factors of PD are of benefit for earlier preventive therapy that might slow down or even prevent the progression of PD, making the identification of biomarkers for early diagnosis important and necessary. Currently, almost all the risk factors are obtained at some time points in which the PD symptoms are already present [14–16], and there is no developing risk factors obtained from completely normal situation to the onset or even advanced stage of PD in individual person to monitor the progress of PD. Animal models, especially the monkey model, are important tools in experimental medical science to better understand the pathogenesis and therapeutic strategies of PD [17]. Up to now, 1-methyl-4-phenyl-1,2,3,6- tetrahydropyridine (MPTP), which is a neurotoxin specifically damage the DA neurons in SN and cause PD-like symptom [18, 19], has emerged unquestionably as a popular chemical reagent to induce PD model [20].

In the present study, MPTP was used to generate progressive PD monkey model with typical motor symptoms. Nine candidate genes including UCHL1, Parkin, PINK1, LRRK2, TRIM24, MUL1, USP30, DJ-1 and SNCA in whole blood samples were analyzed along with PD progress in an aim to identify specific biomarkers for monitor this progress, and diagnose PD at the early stage. The results showed that six genes’ expression (including Parkin, USP30, MUL1, PINK1, LRRK2 and UCHL1) in peripheral blood were significantly expressed before the present of PD symptoms and then changed along with the progression of PD pathogenesis, and the terminal results were consistent with those of in SN. Thus these results could provide an insight into the functions of these genes at different stages of PD, and may facilitate the development of biomarkers for early diagnosis and efficacy evaluation of new drugs on PD model and patients.

## RESULTS

### Behavioral assessment

**Figure 1:**
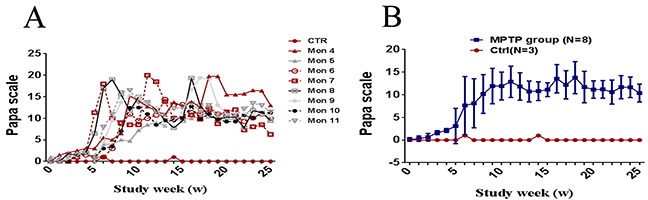
Evolution of motor score by Papa scale **(A)** Longitudinal evolution of the clinical score (in weeks) through the protocol for each monkey. **(B)** Longitudinal evolution of the clinical score (in weeks) through the protocol for all cases. Data are expressed as the mean ± SD.

The monkeys were divided into three groups, the sensitive group (total dose, 15.83±1.47mg/kg, MPTP, N=3), moderate sensitive group (total dose, 25.17±3.38 mg/kg, MPTP, N=3) and hyporesponsiveness group (total dose, 47.05±1.35 mg/kg, MPTP, N=2), according to their personal sensitivity and distinct clinical behavioral symptoms. The sensitive group were first revealed motor abnormalities, while the hyporesponsiveness group were the last. However, with the continued MPTP injection, all the monkeys showed mild PD symptoms at the 9^th^ week and gradually reached a classic and stable parkinsonism stage at the 18^th^ week, since then no significant variation between them were observed (Figure 1). In addition, all the monkeys reproduced classic motor phenotypes, including posture and resting tremor, rigidity, bradykinesia and gesture instability.

### Pathological examination of SN

**Figure 2:**
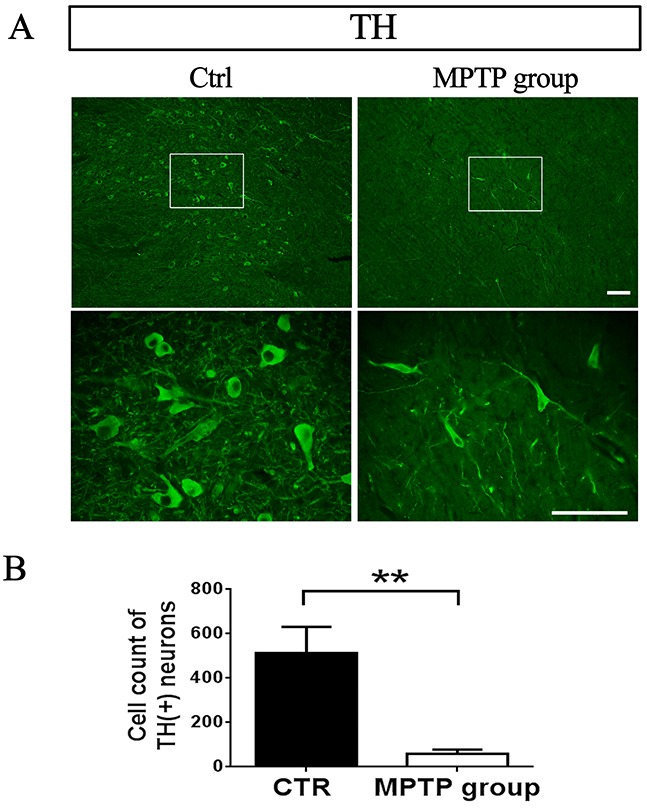
TH-positive neurons in SNpc decreased after MPTP injection **(A)** Representative images from control monkeys injected with saline and PD monkeys injected with MPTP. Scale bar, 100μm. **(B)** DA neurons count in 4x.^**^P=0.000.

Anti-TH immunostaining showed that the number of DA neurons in SN was significantly decreased in PD monkeys (P<0.01, N=8) compared with control group (N=3) (Figure 2). The result indicate that the MPTP-induced monkey model is very successful.

### Changes of mRNA of interested genes in whole blood and SN

To identify the transcriptional profiles associated with PD process, we probed RNA extracted from whole blood of eight monkeys during the intoxication of MPTP. The expressions of UCHL1, Parkin, PINK1, LRRK2, TRIM24, MUL1, USP30, DJ-1 and SNCA mRNA were assessed by RT-qPCR in whole blood.

**Figure 3:**
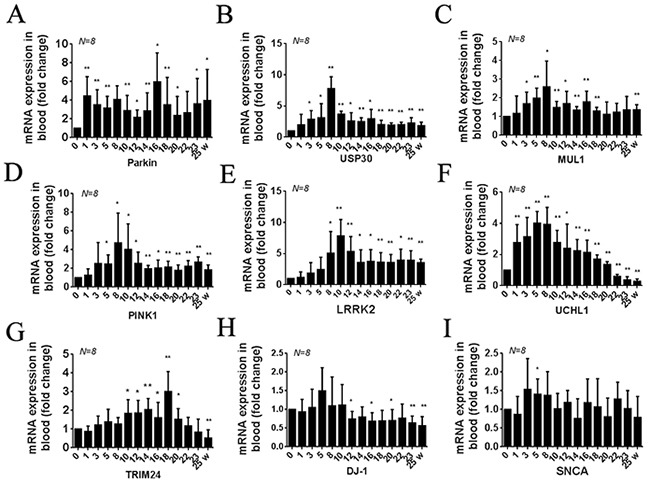
Summary of transcription (RNA) profiles in peripheral blood samples from MPTP-induced monkeys Quantification shows the different times of Parkin **(A)** USP30 **(B)** MUL1 **(C)** PINK1 **(D)** LRRK2 **(E)** UCHL1 **(F)** TRIM24 **(G)** DJ-1 **(H)** and SNCA **(I)** mRNA expression in all monkeys. Data are expressed as mean ± SD. ^*^P < 0.05, ^**^P<0.01, compared with self-control (0W).

Compared with the pro-MPTP period (0W), the expression of Parkin mRNA significantly increased (P<0.01, N=8) at the 1^st^ week of MPTP injection and remained at a high level (P<0.01, N=8) until the end (Figure 3A). The expression of USP30, MUL1, PINK1, and LRRK2 mRNA, significantly increased at the 3^th^, 3^th^, 5^th^, and 8^th^ week and reached to the highest level at the 8^th^, 8^th^, 8^th^, and 10^th^ week (P<0.01, N=8), respectively, and then remained high until the end (Figure 3B, 3C, 3D, 3E).

Compared with the pro-MPTP period (0W), the expression of UCHL1 mRNA significantly increased at the 1^st^ week and reached to the highest level at the 8^th^ week, since then gradually decreased and significantly lower than normal value at the 22^th^ week (P<0.01, N=8) (Figure 3F). The expression of TRIM24 mRNA was slowly increased, significantly increased at the 10^th^ week, and reached to the highest level at the 18^th^ week, then gradually decreased and significantly lower than normal value at the 25^th^ week (Figure 3G).

The expression of DJ-1 mRNA was not significantly changed before the week of ten, then significantly decreased and maintained low until the end (P<0.01, N=8) (Figure 3H). Meanwhile, the expression of SNCA mRNA was not significantly changed excepted at the 5^th^ week (Figure 3I).

The six genes, including UCHL1, Parkin, MUL1, USP 30, PINK1 and LRRK2, changes were all earlier than clinical symptoms.

**Figure 4:**
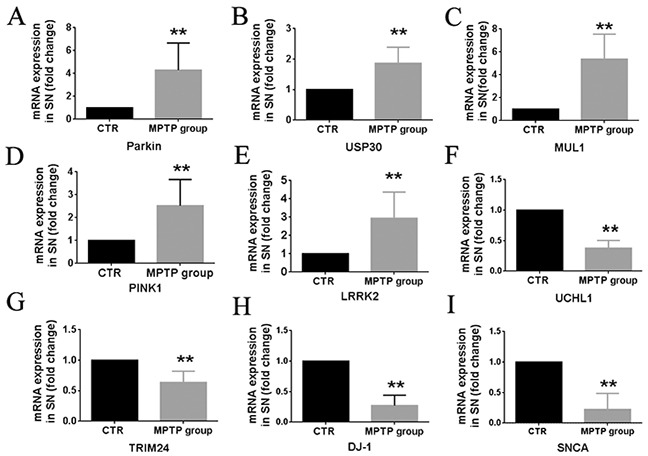
Summary of transcription (RNA) profiles in brain tissues from MPTP-induced monkeys and control monkeys Quantification shows the Parkin **(A)** USP30 **(B)** MUL1 **(C)** PINK1 **(D)** LRRK2 **(E)** UCHL1 **(F)** TRIM24 **(G)** DJ-1 **(H)** and SNCA **(I)** mRNA expression in SN between MPTP group and control group. Data are expressed as mean ± SD. ^*^P < 0.05, ^**^P<0.01, compared with control monkeys.

To investigate the validity of the gene measurements in blood, the genes mRNA expression in SN were also be detected by RT-qPCR. Compared with control group, the expression of UCHL1, TRIM24, DJ-1 and SNCA mRNA in SN was significantly decreased (P<0.01, N=8) (Figures 4F, 4G, 4H, 4I), and the expression of Parkin, USP30, MUL1, PINK1 and LRRK2 mRNA in SN was significantly increased (P<0.01, N=8) (Figure 4A, 4B, 4C, 4D, 4E). The terminal results of whole blood were highly consistent with those of in SN.

## DISCUSSION

PD is a progressive neurodegenerative disorder manifested by a broad spectrum of motor and non-motor features, some of which are present in other parkinsonian syndromes, such as progressive supranuclear palsy (PSP), multiple system atrophy (MSA), and corticobasal degeneration (CBD) [21]. Insufficient of perfect and definitive clinical diagnostic tests make the differentiating PD from related disorders a challenge. Therefore, further researches uncovering disease specific biomarkers allowing for its differentiation from other neurodegenerative disorders are required. Considerable damage occurs before onset of clinical symptoms in PD patients, the identification of biomarkers for the early stage is important. Not only will these biomarkers be useful for diagnosing the disease in affected persons, it will be useful for identifying family members or populations at risk, thus providing an opportunity to initiate neuroprotective therapy at an asymptomatic stage. Literatures have reported the transcription (RNA) profiles in peripheral blood samples might be of diagnostic value for estimating the risk of developing sporadic PD [16, 22]. Mitochondrial dysfunction, reactive oxygen species (ROS), proteasome dysfunction and neuroinflammation are implicated in PD [23–26], as the mutations or changes of genes related with these processes, such as PINK1, Parkin, DJ-1, SNCA, UCHL1, LRRK2, TRIM24, MUL1 and USP30, are found to be widely present in PD [27–29]. Thus, it's reasonable to take these genes as potential markers for the PD diagnosis.

Animal models are important aid to study pathogenesis and therapeutic strategies in human diseases. So far, several chemical materials, including reserpine, methamphetamine, 6-OHDA, MPTP, paraquat-maneb, rotenone, 3-nitrotyrosine and transgenic α-synuclein have been used to generate Parkinson's disease in mice, monkey and some other animals [17]. Among these models, MPTP induced monkey model is probably the most relevant model available that can generate clinical symptoms remarkably similar to sporadic PD in humans. MPTP cross the blood-brain barrier after administration and is metabolized into its active metabolite 1-methyl-4-phenylpyridinium (MPP^+^) in astrocytes. Then MPP^+^ is selectively absorbed into DA neurons *via* dopamine transporter (DAT), and is thus selectively inhibit mitochondrial complex I [30]. In this study, we developed a progressive and stabilized PD monkey model by intramuscularly injection of MPTP for 22 weeks, with no obvious damage on blood biochemistry (detected every one or three weeks, see Supplementary Figure 1), and kidney and liver architecture (detected at the end by hematoxylin- eosin staining, see Supplementary Figure 2), but with a gradual destruction of DA neurons in substantia nigra and a corresponding progressive appearance of PD symptom. And the selected biomarkers were detected in the blood of monkeys at different stages during the PD progress.

Both PINK1 (PTEN-induced putative kinase) and Parkin (E3-ubiquitin ligase) are located in mitochondria, share a common pathway in PD pathogenesis: The clinical symptoms of Parkin-linked parkinsonism could be influenced by PINK1 mutation [31]; Overexpression of Parkin rescues the muscle damage in PINK1-deficient drosophila [32, 33]. MUL1, which function to guard mitochondrial homeostasis and promote fragmentation of mitochondria, acts in parallel with PINK1 and Parkin, suppresses PINK1 or Parkin mutant phenotypes in Drosophila [34]. MUL1, PINK1, and Parkin were obviously increased in stage I (0W-8W), while all monkeys only showed mild PD symptoms. Thus, this results suggested damages to mitochondrial related degradation at this stage. UCHL1 protein is a deubiquitinating enzyme to cleave the carboxy-terminal peptide bond of polyubiquitine chains, and its I93M mutant mice exhibits decreased enzymatic activity and significant loss of dopaminergic cells [35, 36]. USP30, also one mitochondria-localized deubiquitinase enzyme, suppression of which maintaines mitochondrial health, improves climbing capability, and preventes dopamine depletion in PINK1 or Parkin mutant Drosophilia [33]. So, suppression of this gene may be a promising strategy for PD treatment [37]. LRRK2 (PARK8), which is highly expressed throughout the brain in the dopaminoceptive regions instead of dopaminergic neurons, is suggested to be involved in maintaining functions of autophagy and lysosome [38], regulating neurite growth and cytoskeleton dynamics [39], modifying protein translation and vesicle trafficking [35, 40]. So, the three proteasome related genes (UCHL1, USP30, LRRK2) highly expressed in the present study indicate activation of ubiquitin related degradation since stage I, increased UCHL1 and LRRK2 may act as protect role, increased USP30 may act as a damage role, however.

At stage II (8W-16W), all monkeys showed moderate PD symptoms and gradually reached a state of stable parkinsonism. In the state, mitochondria and proteasome related genes stayed increase, while antioxidant gene (DJ-1) was gradually decreased, suggesting an overwhelm of antioxidant defense by increased oxidative stress. At stage III (17W-22W), all monkeys showed classic and stable PD symptoms, meanwhile, the antioxidative gene DJ-1 in blood remained significantly decreased compared with self-control (0W). At the post-MPTP stage (23W-25W), the clinical symptoms of PD were stabled, at which stage the degeneration of DA neurons and neuritis reached to 80-90%, and the expression of interested genes was similar with stage III. It is noteworthy that the mRNA level of SNCA was not significantly elevated (excepted at the 5th week) during the administration of MPTP, which could be explained by that SNCA mainly expresses in brain and the changes of its level in blood was not enough to be sensitively detected by RT-qPCR [41]. However, the SNCA mRNA significantly decreased in SN at last, rather than accumulation, which can be explained by that 90% of DA neurons were already degenerated at last. TRIM24, one of nuclear localization protein involved in the transcription control of some nuclear receptors, has been found reduced in the peripheral blood and skin explants from parkinsonians [42], directly regulates genes in lipid metabolic, inflammation and damage pathways [43], acts negatively as a p53 (repressed by parkin in PD) regulator and leads to p53-induced apoptosis when depletion of TRIM24 in animal model [44–46]. So the changes of TRIM24 expression in the present study can indicate aberrantly regulation of lipid metabolic, inflammation and apoptosis since stage II. Furthermore, the accurate functions of the above genes remain poorly understood and some of the gene products may share a common pathway that protect or damage nigral neurons during the progress of PD. However, in this study, the longitudinal expression profiles can help us know more about the genes associated with sporadic PD and the relationship between each gene.

In conclusion, MPTP as a specific DA neuronal insult can induce classic clinical symptom of PD accompanied with progressive change of PD-related genes in blood, which reflect pathology progress at different stages. So, the genes UCHL1, Parkin, USP30, MUL1 and PINK1 mRNA may act as early biomarkers, and LRRK2, TRIM24, and DJ-1 mRNA in blood may act as late biomarkers, to mirror the disease process, as well as the treatment effects of sporadic PD.

## MATERIALS AND METHODS

### Animals

Eleven rhesus monkeys (Macaca mulatta lasiotis) between 3-4.5 years (young adults) and weighting 3.5-5 kg at the beginning of the experiment were obtained from the Sichuan Primed Biological Technology Co., Ltd / National (Sichuan) Experimental Rhesus Monkey Resources Base (Certificate No SCXK(Chuan): 2013-105). Animals were housed in individual primate cages (one animal per cage) and their care and treatment were in strict accordance with the “the National Institutes of Health Guide for the Care and Use of Laboratory Animal” of the United States, and all the experimental protocols had been reviewed and approved by the Animal Welfare and Use Committee.

### MPTP-induced parkinsonism

At the beginning of the research, eight monkeys from MPTP group were respectively daily administrated with a small dose (0.2 mg/(kg·d)) of 1-methyl-4-phenyl-1,2,3,6- tetrahydropyridine (MPTP-HCl, Product Number: M0896, Sigma, St. Louis, MO), and then accepted the reagent by every 2-4 days to stable the clinical phenotype after appeared typical motor symptoms. Another three monkeys from control group were accepted the injection of saline instead, and the other conditions were the same with MPTP group.

### Behavioral assessment

Before and after two weeks of the experimental protocol, assessment was carried out all day by video tape. The severity of behavioural phenotype of PD was evaluated by using the Papa scale [47, 48]. Briefly, the Papa scale including: posture (0 – 2); gait (0 – 2); tremor (0 – 2); general mobility (0 – 4), hand movements (0 – 2), climbing (0 – 4), holding food (0 – 1), eating (0 – 1), social interaction (0 – 2). Total score can vary from 0 to 20.

### Blood collection

Blood collection was carried out by vein around 8:30-9:00 _AM_ before breakfast every two or three weeks. Then more fresh fruits and vegetables than normally were given to supplemental nutrition.

### Total RNA extraction and complimentary DNA synthesis

Total RNA was isolated from fresh peripheral blood samples by using RNAiso Blood Extractor reagent (Takara Biotechnology Co., Ltd. Dalian, China) according to the manufacturer's instructions. RNA integrity was conducted by 1.2% formaldehyde agarose gel electrophoresis, and the 28S/18S ratio was >1.5. In addition, the concentration of total RNA was measured by spectrophotometer, and the A260/A280 ratio was typically >1.9. Total RNA samples were digested with DNase-I to remove contaminated genomic DNA, and subsequently the standard reverse transcription was performed using the PrimeScript^TM^ RT reagent Kit (Takara Biotechnology Co., Ltd. Dalian, China) and each RT reaction contained 0.5 μg of total RNA according to the manufacturer's instructions, then stored at −70°C for further use.

### Quantitative real-time RT-qPCR analysis

Quantitative real-time RT-qPCR was conducted for 10 genes (Table 1): α-synuclein (SNCA); PTEN-induced putative kinase 1 (PINK1); Parkin; DJ-1; leucine-rich repeat kinase 2 (LRRK2); MUL1; tripartite motif containing 24 (TRIM24); ubiquitin C-terminal hydrolase (UCHL-1); ubiquitin-proteasome system 30 KDa protein (USP30); β-actin (used as house-keeping gene).

**Table 1:** Sequences of primer pairs used for RT-qPCR

Genes	Accession number (Gene Bank)	Primer pairs sequence (5′-3′)	Location (bp)	Size (bp)
SNCA	XM_001095402.2	AGCAAGTGACAAATGTTGGAGGA	152-174	128bp
		ATTCTTGCCCAACTGGTCCTT	259-279	
PINK1	JU325788.1	TCCTCGTTATGAAGAACTATCCCTG	855-879	163bp
		AGGATGTTGTCGGATTTCAGGT	996-1017	
Parkin	XM_001100436.2	TGTGGGTTTGCCTTCTGCC	1078-1096	116bp
		GCGGCTCTTTCATCAACTCTGT	1172-1193	
DJ-1	JV046088.1	GAGCAGAGGAAATGGAGACGG	38-58	139bp
		TCAAGACTGGCATCAGGACAAAT	154-176	
LRRK2	XM_002798570.1	ATGCACTCACGAGCTTTCCAC	286-306	137bp
		AGAGACATCAAGGTTAGCAACACAA	398-422	
MUL1	NM_001261655.1	CTTGCTGTGGACTTGGTGGAG	1410-1430	102bp
		TGCGGCAGAACCTCTTTGG	1493-1511	
TRIM24	NM_001260835.1	ATACCACGACAAGCAATAAAGCC	1636-1658	139bp
		TGGGACTGGAAGGAGTAGAGGAT	1752-1774	
UCHL1	JV044711.1	GCTGCTGTTTCCACTCACGG	150-169	146bp
		CTGCGTGTATAAGTCCGATTGTG	273-295	
USP30	NM_001257829.1	GTTCGATTTGATACCTTTGATAGCC	727-751	120bp
		AACATCCCGCACTGATTCTGA	826-846	
β-actin	NM_001033084.1	GTGACGTGGACATCCGTAAAGAC	854-876	162bp
		CAGAGTACTTGCGCTCAGGAGG	994-1015	

All the RT-qPCR procedures were performed by the Bio-rad CFX96 Sequence Detection System in 25 μl reaction containing 12.5 μl of SYBR®Premix Ex Taq II, 2μl of cDNA, 1 μl of each primer, and 8.5 μl of dH_2_O. Polymerase chain reactions were carried out using the following temperature profile: at 95°C for 3 minutes, 95°C for 10 s, 53-59°C for 30 s, and 72°C for 10 s, 40 cycles, and 72°C for 10min. The relative expression analysis was quantified by the comparative Ct method using the Bio-rad CFX96 system program. Finally, the delta Ct was converted into fold changes. All PCR reactions were run in three duplicates.

### Histopathologic evaluation

At the end of experiment, all monkeys were anaesthetized and their brain were immediately dissected. Left cerebral hemisphere of every monkey was cut into coronal slices (400 μm) fixed in 4% paraformaldehyde for at least 24h, right cerebral hemisphere was cut out the SN stored in liquid nitrogen until use. After continuous coronal slices (15 μm), anti-TH (1:1000, ENZO, USA) immunofluorescence staining was used to observed the degeneration of DA neurons in SN. Then the photographs were captured with fluorescence microscopy (OLYMPUS, BX43, Japan), and the number of DA neurons was counted by Image-Pro Plus 5.1 (USA) in 4×.

### Statistical analysis

Data analysis was performed using Graphpad Prism 5.0, independent sample test was analyzed using SPSS 20.0 software (IBM Corp, USA), and results were expressed as mean ± standard deviation (x ± SD). ^*^P<0.05 and ^**^P<0.01 were considered to be significantly differences compared with self-control (0W) or compared with control monkeys injected with saline.

## SUPPLEMENTARY MATERIALS FIGURES


