# Supplementary material for: Pilot study: molecular risk factors for diagnosing sporadic Parkinson's disease based on gene expression in blood in MPTP-induced rhesus monkeys
Source: Oncotarget. 2017 Nov 10;8(62):105606–14. doi: 10.18632/oncotarget.22348 (PMC5739663; doi:10.18632/oncotarget.22348)
Supplement: Supplementary file 1 [file oncotarget-08-105606-s001.pdf]

Pilot study: molecular risk factors for diagnosing sporadic Parkinson's disease based on gene expression in blood in MPTP-induced rhesus monkeys

SUPPLEMENTARY MATERIALS

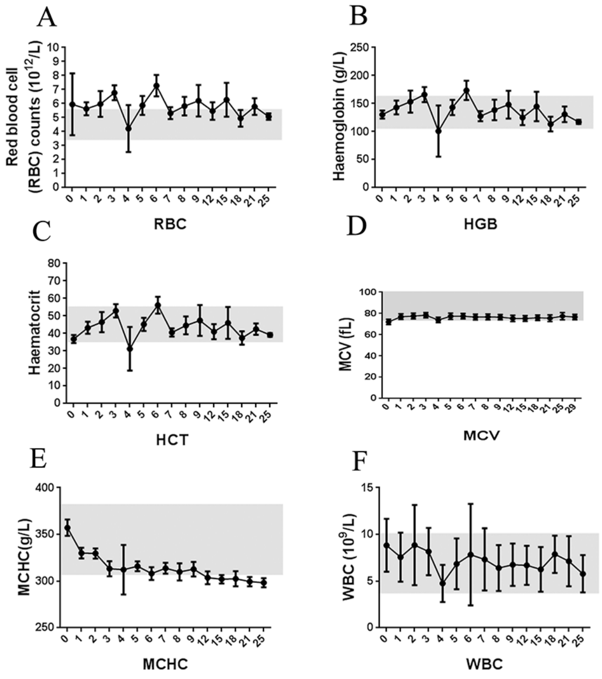

Supplementary Figure 1: Blood biochemistry detected from the beginning to the end. All the factors were in the normal level. Abbreviation: RBC: Red blood cell counts; HGB: Haemoglobin; HCT: Haematocrit; MCV: Mean corpuscular volume; MCHC: Mean corpuscular haemoglobin concentration; WBC: White blood cell counts.

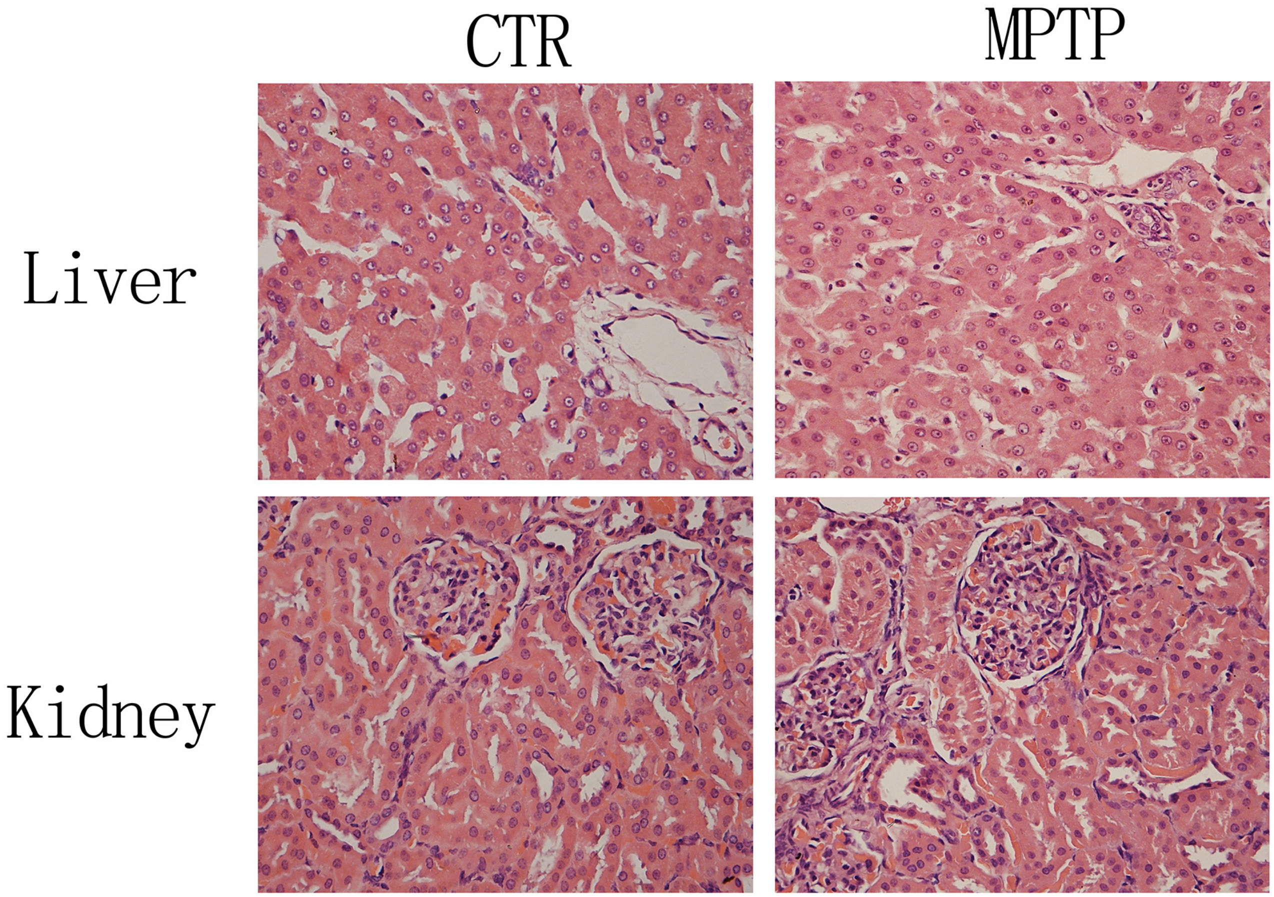

Supplementary Figure 2: Hematoxylin- and eosin-stained liver and kidney sections of monkeys. Both control group and MPTP group showing normal architecture.
